# Should Echolalia Be Considered a Phonic Stereotypy? A Narrative Review

**DOI:** 10.3390/brainsci11070862

**Published:** 2021-06-29

**Authors:** Jacopo Pruccoli, Chiara Spadoni, Alex Orsenigo, Antonia Parmeggiani

**Affiliations:** 1 IRCCS Istituto delle Scienze Neurologiche di Bologna, U.O. Neuropsichiatria dell’Età Pediatrica, 40138 Bologna, Italy; jacopo.pruccoli@studio.unibo.it (J.P.); chiara.spadoni83@gmail.com (C.S.); alex.orsenigo@studio.unibo.it (A.O.); 2 Dipartimento di Scienze Mediche e Chirurgiche, University of Bologna, 40138 Bologna, Italy

**Keywords:** echolalia, autism spectrum disorder, stereotypy, phonic, restrictive and repetitive behaviors

## Abstract

The *Diagnostic and Statistical Manual of Mental Disorders*, fifth edition (DSM-5) defines echolalia as a pathological, parrotlike, and apparently senseless repetition (echoing) of a word or phrase just uttered by another person and classifies this condition among the “restrictive and repetitive behaviours” of Autism Spectrum Disorder (ASD). The authors reviewed the existing literature on echolalia and its role in the development of children with ASD. Current conceptualizations include echolalia among repetitive behaviors and stereotypies and thus interpret this symptom as lacking any communicative significance, with negative effects on learning and sensory processing. Echoic behaviors, however, have been described in neurotypical infants and children as having a substantial effect on the consequent development of language and communication. Relevant research has documented a functional role of echolalia in ASD children as well since it facilitates the acquisition of verbal competencies and affords a higher degree of semantic generalization. This developmental function could be restricted to specific contexts. Considering echolalia as stereotypy and treating it as a disturbing symptom could impair the development of ASD-specific learning and communication processes. In light of this evidence, the authors propose a different conceptualization of echolalia and suggest that this symptom be considered among atypical communication patterns in children with ASD, with implications for treatment and prognosis.

## 1. Introduction

Autism Spectrum Disorder (ASD) is a neurodevelopmental disorder characterized by difficulties in reciprocal social communication and interaction (criterion A) and by repetitive, restricted behaviors and interests (RRBs; criterion B). Frequent comorbidities have been documented between ASD and mental health conditions such as anxiety disorders [1], attention-deficit/hyperactivity disorder [2], and eating disorders [3]. The severity of ASD is recorded as the level of support needed for each of these psychopathological domains [4].

RRBs represent a major clinical element for neuropsychology, diagnosis, and treatment of ASD [5]. RRBs cover a wide range of behaviors. The DSM-5 identifies the following criteria:

1. Stereotyped or repetitive motor movements, use of objects or speech;
2. Insistence on sameness, inflexible adherence to routines, or ritualized patterns of verbal or nonverbal behavior;
3. Highly restricted, fixated interests with abnormal intensity or focus;
4. Hyper- or hypo-reactivity to sensory input or unusual interest in sensory elements.

As for the frequency of specific subtypes of these symptoms, stereotypies were documented as the most frequent early RRBs in a recent study of 105 individuals with ASD [6]. Stereotypies are behaviors that meet the criteria of persistence over time, lack of qualitative variability, immutability with respect to the surrounding environmental changes, and asynchrony with the developmental age of the subject [7]. They are typically classified as motor stereotypies (MS) and phonic stereotypies (PS) [8].

Criterion B1 for ASD in the DSM-5 lists echolalia among stereotyped/repetitive modalities of speech [4]. According to the glossary of technical terms included in the *Diagnostic and Statistical Manual of Mental Disorders*, fifth edition (DSM-5), echolalia can be described as the pathological, parrotlike, and apparently senseless repetition (echoing) of a word or phrase just spoken by another person [4]. Despite its occurrence in a plethora of neurocognitive disorders such as catatonia [9], dementia [10], and intellectual disabilities [11], echolalia is most notably interpreted as an essential clinical sign of ASD since it is one of the key behaviors that contribute to its diagnosis [4].

This article provides a narrative review of the existing literature on the nature of RRBs as signs and symptoms of ASD, focusing particularly on phonic stereotypies, language development, and echolalia. The main reasons and issues behind the current conceptualization of echolalia as a stereotyped and apparently meaningless behavior are explored, and possible alternative theoretical perspectives are discussed.

## 2. Methods

**Figure 1:**
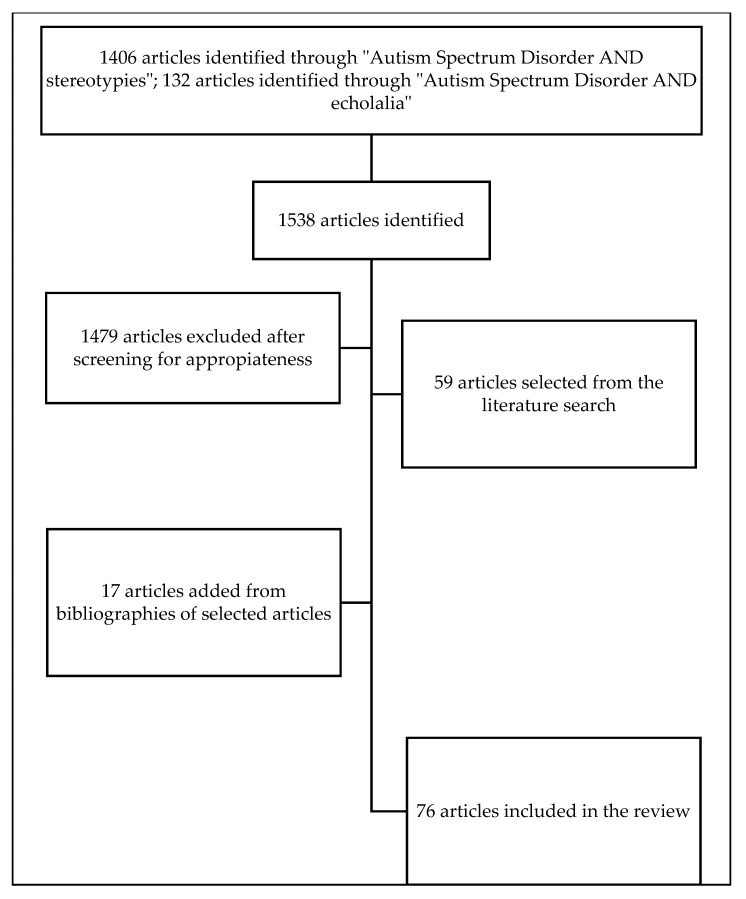
Flowchart of the study.

An extensive web-based search was conducted using PubMed, Web of Science, and Google Scholar. Key search terms included “Autism spectrum disorder AND echolalia” and “Autism spectrum disorder AND stereotypies”, and variations, combined with study filters for original research, case reports, and case series. A total of 1538 articles were identified. All articles were screened manually for content appropriateness. Additional articles were identified from the reference lists of screened papers. Duplicates were removed. At the end of the selection process, a total of 76 articles were included in this review. Only papers published in English were reviewed. The flow chart of the study is shown in Figure 1.

## 3. Results

### 3.1. RRBs and Stereotypies

According to the aforementioned general description, any repetitive, rigid, and invariable action with a tendency to be inappropriate for the context could be classified as a stereotypy [12]. Classically, stereotypies were presumed to serve no specific function [13], and their emergence was regarded as a mere behavioral consequence of altered genetic, developmental, and/or neuroanatomical patterns. Although research in the neurobiological field has provided interesting data regarding the neuroanatomical circuits responsible for the origin of stereotypies [14,15,16,17], a further stream of studies has simultaneously explored the neurobehavioral mechanisms that seem to underlie the emergence and persistence of such behaviors. Lovaas and collaborators were the first to propose an operant model for stereotypies claiming that such behaviors, despite being the result of unclear and unidentified biological factors, might produce perceptive consequences capable of reinforcing the stereotyped action [18]. Subsequent independent research supported this model [19,20,21]. Permitting or denying access to the sensorial output of the stereotypy does, in fact, reduce or increase, respectively, the undertaking of such behavior [22,23,24,25,26,27]. Alongside the abovementioned auto-stimulatory mechanism and its plausible role in sensory modulation [28], stereotyped behaviors may be influenced by factors other than the stereotypy itself [29]; for example, positive and negative social interactions [30]. Moreover, although some research shows that stereotyped behaviors may interfere with learning processes [31] and sensory processing [32], other evidence suggests that PS, as opposed to MS, may facilitate the acquisition of new social and behavioral skills [33].

### 3.2. Phonic Stereotypies

Phonic stereotypies are fixed, chronic, and non-propositional acoustic behaviors [34]. PS may vary from repetition of simple vocalizations to the purposeless emission of more articulated sounds, such as words or phrases [35]. Despite the clinical relevance played by such behaviors in the identification and management of ASD individuals, the literature presents just a few studies on this topic [7,36]. Moreover, the current scientific literature lacks a validated classification. Independent attempts were made to categorize PS focusing on different distinctive features, such as the complexity or the quality of the sound emission. We summarize below the two classifications of PS in the literature.

Classification of PS based on the complexity of phonation [34]:

1. Simple: any straightforward and distinct sound whose utterance requires little effort;
2. Complex: any sound that mimics physiological functions such as hyperventilation, nonsensical word repetitions, or any other sound that is notably complex in nature. Classification of PS based on the quality of phonation:
3. Vocal: communicative vocalizations, syllables, echolalia [37];
4. Non-vocal: guttural emissions and non-communicative vocalizations such as throat clearing, grunting, buzzing, whistling, laughing, and coughing [38].

The lack of a unanimous classification makes it difficult to obtain definitive data on the prevalence and impact of such behaviors both in ASD individuals and neurotypical children, a population that may still manifest stereotyped behaviors [39]. An accurate categorization would serve an important clinical purpose, as atypical vocal production is considered an early diagnostic marker for ASD [40].

### 3.3. Language Development in Children with ASD

Even though atypical early vocalization is not pathognomonic for ASD, its manifestation represents the first reason why ASD children access health services [40]. A series of relevant differences emerge when comparing atypical phonic production in children with ASD and canonical babbling in neurotypical children. Canonical babbling is an essential phase of normal language development; it is characterized by the rapid reduplication of syllables with at least one vowel-like element and one supraglottal consonant-like element [41]. Since it is a highly conserved evolutionary stage in humans [42,43], it was suggested that it is of vital importance for the subsequent development of spoken language [44]. The frequency of this specific vocalization and the variety of spoken consonants are predictive of expressive language production in neurotypical individuals [45], whereas altered production patterns may be observed in a variety of conditions such as deafness, Rett Syndrome, X-Fragile Syndrome, and ASD [46]. Starting from the second semester of life, ASD individuals tend to produce a decreased number of canonical syllables and fewer middle and late consonants. Atypical vocalization, instead, is more frequent in ASD individuals than in neurotypical individuals [47]. Considering that babbling elicits parental responses more efficiently than pre-linguistic vocalizations [48], it was suggested that the altered vocal production leads to a reduced frequency of communicative interactions with the parents, which compromises the social reinforcement of vocal exploration. Swanson and collaborators describe that infants with a high familial risk for ASD were found to be hyper-vocal compared to the control group; however, their phonic production was not aimed at establishing social interactions [49,50]. On a similar note, it was suggested that the cry of ASD individuals might play a negative role in their linguistic development. In fact, when compared to their neurotypical counterpart, ASD infants are more prone to crying at a higher fundamental frequency and with shorter inspiration pauses [51]. The difference in sound quality, which is associated with conditions of poor wellbeing and disease in neurotypical newborns and infants [52], elicits a response of greater distress in the parents. This, combined with a higher degree of the frustration associated with parents’ inadequate interpretation of children’s needs [53,54], negatively affects the parent-child interaction, possibly contributing to the impairment of the communicative and linguistic functions and the development and persistence of verbal and non-verbal stereotyped behaviors in the child [36].

### 3.4. Echolalia and Echoic Behaviour

Despite being included in the same category and sharing many characteristics with other phonic stereotypies, echolalia shows distinctive traits. A proper characterization of echolalia should be based on an exact distinction between echolalia in children with ASD and echoic behaviors in non-ASD children [55].

Echoic behavior is commonly found in neurotypical children below the age of 30 months [55,56] and is widely speculated to be a normal phase of language development [55,56,57,58,59]. The repetition of phonic elements directly increases as more verbal stimuli are presented to the child, thus facilitating the learning process. Echophenomena may therefore represent an active approach to language comprehension and vocal production [59,60,61]. Early research on the subject conjectured that in ASD children, echolalia was functionally the same as echoic behavior in non-ASD individuals [62]. However, neurotypical echoic behavior differs considerably in terms of quality and frequency from echolalia as uttered by children with ASD [63]. Children with ASD tend to repeat words or sentences in a parrotlike fashion, whereas neurotypical children extract crucial morphemes but rearrange the rest of the sentence from a grammatical point of view [64]. Described as such, these formal differences are not representative of echolalia as a purposeless behavior since the content of the repetition and the act of repetition itself very rarely coincide [65]. In fact, according to research aimed at identifying the functional value of immediate echolalia in ASD individuals by focusing on simultaneous paralinguistic indicators, only 4% of the 1009 observed echolalic behaviors were deemed purposeless. The following functional classes were recognized: dialogic, declaratory, reiterative, affirmative, demanding, and self-regulatory [66]. The authors arrived at this categorization by videotaping five ASD children in multiple contexts (i.e., home, interactions with a teacher, interactions with classmates). To describe echolalia, they considered three factors, namely communication context, structural characteristics and latency of onset, and recorded behavioral features (gaze direction, pointing, touching, general body orientation and movement and manipulation of toys or objects) as well as linguistic features (structural changes in the echoic utterances; intonation and latency of the utterance) [66].

Six behavioral and linguistic core attributes of different echolalic responses were identified: evidence of attention, echo directed/not directed to a person, degree of change, the timing of change, evidence of comprehension, the expectation of a response from an adult. According to this framework, seven functional categories of echolalic responses were identified: (1) Non-focused (non-directed to person; no evidence of behavioral modulation; rigid and stereotyped); (2) Turn-taking (evidence of attention and directedness to person; no evidence of behavioral change; rigid); (3) Declarative (evidence of attention and directedness to person; demonstrative gestures; variably rigid and stereotyped); (4) Rehearsal (non-directed to the interlocutor; a verbal or nonverbal response is initiated subsequent to echolalia by the subject: “thinking out loud”); (5) Self-regulatory (non-directed to the interlocutor; helping the child to direct their own behavior; “Go find the dog”); (6) Yes-Answer (directed to the interlocutor; evidence of affirmation related to echolalia: “affirmation by repetition”); (7) Request (directed to the interlocutor; the desire to obtain an object or perform an action).

It was also hypothesized that the number of functions attributed to immediate echolalia might vary according to the linguistic competence of ASD children [66]. Echolalia may be an advantageous communicative strategy for individuals with an intermediate level of verbal capacity, but its usefulness and relative frequency decrease as the child develops more effective communicative tools [67]. Moreover, as for echoic behavior in neurotypical children, allowing a certain amount of echolalia during linguistic tasks was demonstrated to facilitate the acquisition of verbal competencies, affording a higher degree of semantic generalization, especially when the child is examined in a novel setting [68]. A lack of familiarity with the environment is, in fact, associated with the increased frequency of verbal echophenomena [69], which supports the hypothesis that echolalia may be a primary linguistic strategy for ASD children with limited access to alternative communicative skills. This data could have relevant implications for ASD children with limited development of structured language since impaired communication skills represent a key feature of ASD regardless of the verbal skills of the child. Even verbal autistic children show, to some extent, impairment of expressive and receptive pragmatic functions [4].

The context of the communicative act is of crucial importance, as it can significantly influence the responses of ASD children. In fact, these vary in terms of adequacy and repetitiveness depending on the appropriateness of the linguistic expressions uttered by the adult interlocutor [70]. Specifically, it was observed that high constraint utterances, which limit the syntactic and semantic content of the answer (such as test questions or directive commands) are capable of eliciting a greater number of responses, including echolalias, when compared to low constraint utterances (such as spontaneous declarative comments and feedbacks for verbalizations). Some researchers speculated that this is indicative of a compensatory mechanism that ASD children would make use of to reduce the cognitive effort in highly constraining settings [71]. On a similar note, socio-linguistic inputs consisting of unknown words expressed with a high degree of directiveness, such as commands, produced a greater amount of echolalic responses, which suggests that ASD children are not only sensitive to the content of the interaction but also to the communicative style used by the interlocutor [72]. It is interesting to note how ASD children are therefore capable of configuring echolalia (by definition a repetitive and invariable behavior) in different ways, depending on the different interactive environment.

## 4. Discussion

Echolalia is included among the “restrictive and repetitive behaviours” criterion B1 for ASD [4]. This definition results from a series of classical studies on individuals with ASD, which consider repetitive behaviors and stereotypies as being directed to no specific function [13], with negative effects on sensory processing [18] and the learning process [31]. Although echolalia may be typically observed in individuals with ASD or other neurodevelopmental disorders, echoic behaviors were documented to play relevant roles in the development of normal communication in non-ASD children as well [56,59]. Evidence has challenged the supposed meaninglessness of echolalia. Moreover, like echoic behaviors in neurotypical children, echolalia could play an adaptive role in the development of communication skills in children with ASD by facilitating the acquisition of verbal competencies and by affording a higher degree of semantic generalization [66,67]. The adaptive and evolutionary role of echolalia, however, could be restricted to specific developmental levels, such as low degrees of verbal capacities [67] and settings such as novel environments [68].

Echolalia in children with ASD appears to have specific functional qualities, which set it apart from other stereotypies. Whereas RRBs were traditionally considered as interfering factors in learning new skills [73,74], echolalic behavior in ASD children could represent an active strategy for developing an alternative communicative style, allowing social interactions and the exploration of the outer world [75]. Studies in pragmatics have long been emphasizing the importance of non-verbal behaviors, paralinguistic features, and the situational context for the full understanding of language usage and communication. Prizant and collaborators were the first to propose a change in the classical conceptual framework for echolalia: by focusing on the communicative setting and response characteristics (e.g., if the child was addressed directly; whether they changed their behavior at the time of the echolalic utterance; what kind of change they produced and at what time; gaze behavior prior, during and after the response and the functional appropriateness of the utterance to the task), they derived seven functional categories for echolalic responses [66]. Other studies lent support to this classification, albeit lukewarmly [67]. Sterponi and Sharkey subsequently proposed to rethink the traditional perspectives on communication and language in ASD, advocating a multidimensional view on the subject. According to them, language is not simply a referential system reflecting the cognitive level of the individual, but rather an interactional accomplishment, a social action, and a mode for experiencing reality in its entirety [75].

Stereotyped behaviors such as the majority of currently recognized RRBs negatively affect children with ASD [73], contributing to social and learning impairment, and are therefore worthy of early treatments for reduction and resolution [74]. On the other hand, considering every instance of echolalia as a mere RRB could prove counter-productive when developing treatment plans. It is important to thoroughly evaluate the conditions in which echolalia occurs by focusing on behavioral and paralinguistic features before establishing that it has no communicative value [76], for the suppression of a purposeful behavior would deprive ASD-affected individuals of a potentially useful interactive tool.

For the abovementioned reasons, we suggest that the inclusion of echolalia in the “RRB” criterion B1 for ASD is only partially appropriate and that echolalia should not be considered as a mere phonic stereotypy. The potential social and communicative function of echolalia sets it apart from other kinds of stereotypies and makes it more akin to atypical social communication features as listed in the diagnostic criterion A [4]. However, even criterion A1 (“deficits in social-emotional reciprocity”) does not provide a sufficiently specific or accurate description for the inclusion of echolalia. Whether echolalia could properly fit in this category mainly depends on semantic interpretation and the functional values attributed to such behaviors.

## 5. Conclusions

To date, a number of questions regarding echolalia and its functional role in ASD individuals remain unanswered. A change in the conceptualization of this peculiar phonic feature may represent the starting point for future studies searching for more effective therapeutic interventions for ASD children. The thorough categorization of paralinguistic and behavioral features accompanying echolalia would provide specialists with useful tools for determining whether a specific phonic behavior is to be discouraged or could be taken advantage of for the development of alternative communicative strategies.

## Data Availability

Not applicable.

## References

[1] Simonoff E., Pickles A., Charman T., Chandler S., Loucas T., Baird G. (2008). Psychiatric Disorders in Children With Autism Spectrum Disorders: Prevalence, Comorbidity, and Associated Factors in a Population-Derived Sample. J. Am. Acad. Child Adolesc. Psychiatry.

[2] Taurines R., Schwenck C., Westerwald E., Sachse M., Siniatchkin M., Freitag C. (2012). ADHD and autism: Differential diagnosis or overlapping traits? A selective review. ADHD Atten. Deficit Hyperact. Disord..

[3] Pruccoli J., Solari A., Terenzi L., Malaspina E., Angotti M., Pignataro V., Gualandi P., Sacrato L., Cordelli D.M., Franzoni E. (2021). Autism spectrum disorder and anorexia nervosa: An Italian prospective study. Ital. J. Pediatr..

[4] American Psychiatric Association (2013). Diagnostic and Statistical Manual of Mental Disorders, (DSM-V).

[5] D’Cruz A.-M., Ragozzino M., Mosconi M.W., Shrestha S., Cook E.H., Sweeney J.A. (2013). Reduced behavioral flexibility in autism spectrum disorders. Neuropsychology.

[6] Parmeggiani A., Corinaldesi A., Posar A. (2019). Early features of autism spectrum disorder: A cross-sectional study. Ital. J. Pediatr..

[7] Berkson G. (1983). Repetitive stereotyped behaviors. Am. J. Ment. Defic..

[8] Lanzarini E., Pruccoli J., Grimandi I., Spadoni C., Angotti M., Pignataro V., Sacrato L., Franzoni E., Parmeggiani A. (2021). Phonic and Motor Stereotypies in Autism Spectrum Disorder: Video Analysis and Neurological Characterization. Brain Sci..

[9] Burrow J.P., Spurling B.C., Marwaha R. (2021). Catatonia.

[10] Mendez M.F., Perryman K.M. (2002). Neuropsychiatric features of frontotemporal dementia: Evaluation of consensus criteria and review. J. Neuropsychiatry Clin. Neurosci..

[11] Natasha Marrus M.D., Lacey Hall M.S. (2017). Intellectual Disability and Language Disorder. Child Adolesc. Psychiatr. Clin. N. Am..

[12] Turner M. (1999). Annotation: Repetitive Behaviour in Autism: A Review of Psychological Research. J. Child Psychol. Psychiatry.

[13] Birdwhistell R.L., Hutt S.J., Hutt C. (1972). Direct Observation and Measurement of Behavior. Springfield, Ill: Charles C Thomas, 1970. Pp. 224 + xii. $14.95. Am. J. Clin. Hypn..

[14] Lewis F.M., Murdoch B.E., Woodyatt G.C. (2007). Communicative Competence and Metalinguistic Ability: Performance by Children and Adults with Autism Spectrum Disorder. J. Autism Dev. Disord..

[15] Cheung C., Chua S., Cheung V., Khong P., Tai K., Wong T., Ho T., McAlonan G. (2009). White matter fractional anisotrophy differences and correlates of diagnostic symptoms in autism. J. Child Psychol. Psychiatry.

[16] Weng S.-J., Wiggins J.L., Peltier S.J., Carrasco M., Risi S., Lord C., Monk C.S. (2010). Alterations of resting state functional connectivity in the default network in adolescents with autism spectrum disorders. Brain Res..

[17] Uddin L.Q., Esupekar K., Emenon V. (2013). Reconceptualizing functional brain connectivity in autism from a developmental perspective. Front. Hum. Neurosci..

[18] Lovaas O.I. (1987). Behavioral treatment and normal educational and intellectual functioning in young autistic children. J. Consult. Clin. Psychol..

[19] Kennedy G.S.C.H., Souza G. (1995). Functional Analysis and Treatment of Eye Poking. J. Appl. Behav. Anal..

[20] Rapp J.T., Miltenberger R.G., Galensky T.L., Ellingson S.A., Long E.S. (1999). A Functional Analysis of Hair Pulling. J. Appl. Behav. Anal..

[21] Iwata B.A. (2006). On the distinction between positive and negative reinforcement. Behav. Anal..

[22] Rincover A. (1978). Sensory Extinction: A procedure for eliminating self-stimulatory behavior in developmentally disabled children. J. Abnorm. Child Psychol..

[23] Sprague J., Holland K., Thomas K. (1997). The effect of noncontingent sensory reinforcement, contingent sensory reinforcement, and response interruption on stereotypical and self-injurious behavior. Res. Dev. Disabil..

[24] Piazza C.C., Adelinis J.D., Hanley G.P., Goh H.-L., Delia M.D. (2000). An Evaluation of The Effects of Matched Stimuli on Behaviors Maintained by Automatic Reinforcement. J. Appl. Behav. Anal..

[25] Tang J.-C., Patterson T.G., Kennedy C.H. (2003). Identifying specific sensory modalities maintaining the stereotypy of students with multiple profound disabilities. Res. Dev. Disabil..

[26] Higbee T.S., Chang S.-M., Endicott K. (2005). Noncontingent access to preferred sensory stimuli as a treatment for automatically reinforced stereotypy. Behav. Interv..

[27] Fetta A., Carati E., Moneti L., Pignataro V., Angotti M., Bardasi M., Cordelli D., Franzoni E., Parmeggiani A. (2021). Relationship between Sensory Alterations and Repetitive Behaviours in Children with Autism Spectrum Disorders: A Parents’ Questionnaire Based Study. Brain Sci..

[28] Tröster H., Brambring M., Beelmann A. (1991). The age dependence of stereotyped behaviours in blind infants and preschoolers. Child Care Heal. Dev..

[29] Kennedy C.H., Meyer K.A., Knowles T., Shukla S. (2000). Analyzing the multiple functions of stereotypical behavior for students with autism: Implications for assessment and treatment. J. Appl. Behav. Anal..

[30] Durand V.M., Carr E.G. (1987). Social Influences on “Self-Stimulatory” Behavior: Analysis and Treatment Application. J. Appl. Behav. Anal..

[31] Koegel R.L., Covert A. (1972). The relationship of self-stimulation to learning in autistic children1. J. Appl. Behav. Anal..

[32] Lovaas O., Litrownik A., Mann R. (1971). Response latencies to auditory stimuli in autistic children engaged in self-stimulatory behavior. Behav. Res. Ther..

[33] Sherer M.R., Schreibman L. (2005). Individual Behavioral Profiles and Predictors of Treatment Effectiveness for Children With Autism. J. Consult. Clin. Psychol..

[34] Shore L.M., Randel A.E., Chung B.G., Dean M.A., Ehrhart K.H., Singh G. (2010). Inclusion and Diversity in Work Groups: A Review and Model for Future Research. J. Manag..

[35] Van Santen J.P.H., Sproat R.W., Hill A.P. (2013). Quantifying Repetitive Speech in Autism Spectrum Disorders and Language Impairment. Autism Res..

[36] Yankowitz L.D., Schultz R.T., Parish-Morris J. (2019). Pre- and Paralinguistic Vocal Production in ASD: Birth Through School Age. Curr. Psychiatry Rep..

[37] Healy O., Lydon S., Brady T., Rispoli M., Holloway J., Neely L., Grey I. (2018). The Use of Differential Reinforcement of Other Behaviours to Establish Inhibitory Stimulus Control for the Management of Vocal Stereotypy in Children with Autism. Dev. Neurorehabilit..

[38] Min C.-H., Fetzner J. (2018). Vocal Stereotypy Detection: An Initial Step to Understanding Emotions of Children with Autism Spectrum Disorder. Annu. Int. Conf. IEEE Eng. Med. Biol. Soc. (EMBC).

[39] Singer H.S. (2009). Motor Stereotypies. Semin. Pediatr. Neurol..

[40] Herlihy L., Knoch K., Vibert B., Fein D. (2013). Parents’ first concerns about toddlers with autism spectrum disorder: Effect of sibling status. Autism.

[41] Lang S., Bartl-Pokorny K.D., Pokorny F.B., Garrido D., Mani N., Fox-Boyer A.V., Zhang D., Marschik P.B. (2019). Canonical Babbling: A Marker for Earlier Identification of Late Detected Developmental Disorders?. Curr. Dev. Disord. Rep..

[42] Eilers R.E., Oller D.K., Levine S., Basinger D., Lynch M.P., Urbano R. (1993). The role of prematurity and socioeconomic status in the onset of canonical babbling in infants. Infant Behav. Dev..

[43] Oller D.K., Eilers R.E., Basinger D., Steffens M.L., Urbano R. (1995). Extreme poverty and the development of precursors to the speech capacity. First Lang..

[44] Patten E., Belardi K., Baranek G., Watson L.R., Labban J.D., Oller D.K. (2014). Vocal Patterns in Infants with Autism Spectrum Disorder: Canonical Babbling Status and Vocalization Frequency. J. Autism Dev. Disord..

[45] Watt N., Wetherby A., Shumway S. (2006). Prelinguistic Predictors of Language Outcome at 3 Years of Age. J. Speech Lang. Hear. Res..

[46] Marschik P.B., Einspieler C., Sigafoos J. (2012). Contributing to the early detection of Rett syndrome: The potential role of auditory Gestalt perception. Res. Dev. Disabil..

[47] Paul R., Fuerst Y., Ramsay G., Chawarska K., Klin A. (2010). Out of the mouths of babes: Vocal production in infant siblings of children with ASD. J. Child Psychol. Psychiatry.

[48] Warlaumont A.S., Richards J.A., Gilkerson J., Oller D.K. (2014). A Social Feedback Loop for Speech Development and Its Reduction in Autism. Psychol. Sci..

[49] Swanson M.R., Shen M.D., Wolff J.J., Boyd B., Clements M., Rehg J., Elison J.T., Paterson S., Parish-Morris J., Chappell J.C. (2018). Naturalistic Language Recordings Reveal “Hypervocal” Infants at High Familial Risk for Autism. Child Dev..

[50] Warren S.F., Gilkerson J., Richards J.A., Oller D.K., Xu D., Yapanel U., Gray S. (2009). What Automated Vocal Analysis Reveals About the Vocal Production and Language Learning Environment of Young Children with Autism. J. Autism Dev. Disord..

[51] English M.S., Tenenbaum E.J., Levine T.P., Lester B.M., Sheinkopf S.J. (2018). Perception of Cry Characteristics in 1-Month-Old Infants Later Diagnosed with Autism Spectrum Disorder. J. Autism Dev. Disord..

[52] Furlow F. (1997). Human Neonatal Cry Quality as an honest signal of fitness. Evol. Hum. Behav..

[53] Esposito G., Venuti P. (2010). Developmental changes in the fundamental frequency (f0) of infants’ cries: A study of children with Autism Spectrum Disorder. Early Child Dev. Care.

[54] Bornstein M., Costlow K., Truzzi A., Esposito G. (2016). Categorizing the cries of infants with ASD versus typically developing infants: A study of adult accuracy and reaction time. Res. Autism Spectr. Disord..

[55] van Riper C. (1963). Speech Correction.

[56] Nakanishi Y., Owada K. (1973). Echoic utterances of children between the ages of one and three years. J. Verbal Learn. Verbal Behav..

[57] Fay W.H. (1969). On the basis of autistic echolalia. J. Commun. Disord..

[58] Haworth M.R., Menolascino F.J. (1968). Some Aspects of Psychotic Behavior in Young Children. Arch. Gen. Psychiatry.

[59] Esch J.W., Mahoney A.M., Kestner K.M., LaLonde K.B., Esch B.E. (2013). Echoic and Self-Echoic Responses in Children. Anal. Verbal Behav..

[60] Fay W.H., Butler B.V. (1968). Echolalia, IQ, and the Developmental Dichotomy of Speech and Language Systems. J. Speech Hear. Res..

[61] Fay W.H., Butler B.V. (1971). Echo-Reaction as an Approach to Semantic Resolution. J. Speech Hear. Res..

[62] Philips G.M., Dyer C. (1977). Late Onset Echolalia in Autism and Allied Disorders. Int. J. Lang. Commun. Disord..

[63] Charlop M.H. (1983). The effects of echolalia on acquisition and generalization of receptive labeling in autistic children. J. Appl. Behav. Anal..

[64] Sullivan M.T. (2002). Comunicative Functions of Echolalia in Children with Autism: Assessment and Treatment.

[65] Prizant B.M. (1983). Language Acquisition and Communicative Behavior in Autism. J. Speech Hear. Disord..

[66] Prizant B.M., Duchan J.F. (1981). The Functions of Immediate Echolalia in Autistic Children. J. Speech Hear. Disord..

[67] McEvoy R.E., Loveland K.A., Landry S.H. (1988). The functions of immediate echolalia in autistic children: A developmental perspective. J. Autism Dev. Disord..

[68] Charlop M.H. (1986). Setting effects on the occurrence of autistic children’s immediate echolalia. J. Autism Dev. Disord..

[69] Resnick S.M. (1973). A Review of the Literature and an Experimental Analysis of Echolalia.

[70] Curcio F., Paccia J. (1987). Conversations with autistic children: Contingent relationships between features of adult input and children’s response adequacy. J. Autism Dev. Disord..

[71] Rydell P.J., Mirenda P. (1991). The effects of two levels of linguistic constraint on echolalia and generative language production in children with autism. J. Autism Dev. Disord..

[72] Violette J., Swisher L. (1992). Echolalic Responses by a Child With Autism to Four Experimental Conditions of Sociolinguistic Input. J. Speech, Lang. Hear. Res..

[73] Matson J.L., Dempsey T., Fodstad J.C. (2009). Stereotypies and repetitive/restrictive behaviours in infants with autism and pervasive developmental disorder. Dev. Neurorehabilit..

[74] Ghanizadeh A. (2010). Clinical Approach to Motor Stereotypies in Autistic Children. Iran. J. Pediatr..

[75] Sterponi L., De Kirby K., Shankey J. (2014). Rethinking language in autism. Autism.

[76] Saad A.G.D.F., Goldfeld M. (2009). A ecolalia no desenvolvimento da linguagem de pessoas autistas: Uma revisão bibliográfica. Pró-Fono Rev. de Atualização Científica.

